# Folate, Vitamin B6 and Vitamin B12 Intake in Relation to Hyperuricemia

**DOI:** 10.3390/jcm7080210

**Published:** 2018-08-11

**Authors:** Yiying Zhang, Hongbin Qiu

**Affiliations:** 1 School of Public Health, Jiamusi University, Jiamusi 154007, China; yiyingzhang@hrbmu.edu.cn; 2 Department of Epidemiology, School of Public Health, Harbin Medical University, Harbin 150081, China

**Keywords:** hyperuricemia, folate, vitamin B6, vitamin B12, NHANES

## Abstract

To assess the association between intake of folate, vitamin B6, and vitamin B12 with hyperuricemia (HU) among adults from the United States (US), we extracted relevant data from 24,975 US adults aged 20–85 years from the National Health and Nutrition Examination Survey (NHANES) in 2001–2014. All dietary intake was evaluated by 24-h dietary recalls. Multivariable logistic regression analysis was performed to explore the associations after adjustment for confounders. Compared to the lowest quintile (Q1), for males, adjusted odds ratios (ORs) of HU in Q2 to Q5 of folate (dietary folate equivalent, DFE) intake were 0.84 (95% CI, 0.73–0.96), 0.84 (0.73–0.97), 0.72 (0.62–0.84), and 0.64 (0.53–0.77), respectively (*p* for trend <0.0001). In females, adjusted ORs in Q2 to Q4 of folate (DFE) intake were 0.84 (95% CI, 0.71–0.99), 0.81 (0.68–0.96), and 0.82 (0.68–0.99), with a *p* for trend of 0.1475. Our findings indicated the intakes of total folate, folic acid, food folate, folate (DFE), vitamin B12, but not vitamin B6, were inversely related to the risk of HU in males. A lower risk of HU with higher intakes of total folate, food folate, and folate (DFE) was found in females, but with no association between intakes of folic acid, vitamin B6, B12, and the risk of HU for females.

## 1. Introduction

Hyperuricemia (HU) is a metabolic disorder, with a prevalence of over 21% in American adults [1], and in recent decades, has become a serious public health problem. HU is considered to be an essential risk factor for gout arthritis, cardiovascular diseases, type 2 diabetes, hypertension, and chronic kidney disease [2,3,4,5,6,7]. The pathophysiology of HU has not yet been totally explicated.

Folic acid and its derivatives have been reported to inactivate xanthine oxidoreductase enzyme (XOR), which is responsible for the oxidation from hypoxanthine to xanthine, and from xanthine to uric acid (UA) [8,9]. Several studies have suggested a significant association between hyperhomocysteinemia (HHcy) and HU [10,11,12], and HHcy was highly prevalent in gout patients [13]. A lower level of homocysteine (Hcy) has been reported to be associated with a lower risk of HU [14,15]. Previous studies have also demonstrated that reducing Hcy levels can be obtained by increasing intakes of folate, vitamin B6, and vitamin B12 [16]. A randomized, double-blind, actively controlled trial showed that folic acid therapy could lower serum UA concentrations among hypertensive adults [17]. A previous case-control study in Taiwan showed that high folate intake may protect against gout [18]. One study suggested that high doses of supplemental folate may be beneficial in preventing gout and recurrent attacks [19]. However, other studies that used folic acid supplementation failed to lower blood UA concentrations [20,21]. These findings were all from small sample sizes or short treatment durations, and no studies have investigated the relationships between folic acid and serum UA using a large sample size.

To date, no known studies have explored the association between intakes of folate, vitamin B6, vitamin B12, and HU. Therefore, the objective of this cross-sectional study was to investigate this correlation using a large a nationally representative sample in the United States (US), with the hypothesis that intakes of folate, vitamin B6, and vitamin B12 are inversely associated with HU.

## 2. Materials and Methods

### 2.1. Study Populations

Data from National Health and Nutrition Examination Survey (NHANES) represented the total civilian, non-institutionalized population of the US. NHANES is an ongoing, continuous survey, with data publicly released every two years. In each survey cycle, a nationally representative sample is selected using a complex, stratified, multistage probability cluster sampling design. NHANES is administered by the Centers for Disease Control and Prevention (CDC) [22]. NHANES is a publicly available dataset. The data for these surveys including interviews, physical and laboratory examination can be downloaded from the NHANES website (http://www.cdc.gov/nchs/nhanes.htm). We have used data only from the NHANES database in this study. NHANES protocols were approved by the National Center for Health Statistics Research ethics review board, written informed consent was obtained for all participants [23], and additional Institutional Review Board approval for the secondary analyses was not required [24].

A total of 37,215 adults aged 20–85 years provided UA samples for NHANES 2001–2014. Excluded were pregnant women (*n* = 1507); participants with missing or incomplete essential information on demographic, examination, or dietary recall data (*n* = 8912); those taking medications that might affect UA metabolism, such as furosemide, losartan, and allopurinol (*n* = 1420); and those with serum creatinine >1.5 mg/dL [25] were also excluded for consideration of renal dysfunction (*n* = 401). After these exclusions, the total subjects for our study included 24,975 adults (12,218 women, 12,757 men).

### 2.2. Study Variables

All participants were interviewed by the first 24-h dietary recall method to estimate quantitative food and total nutrient intakes through interview questions from 2001 to 2014, and a part of the adult participants participated in second dietary surveys through the telephone interviews 3 to 10 days after the initial recall interview since 2003. We only used the first 24-h dietary recall to obtain total nutrient intakes, including the intakes of total folate, food folate, folic acid, vitamin B6, vitamin B12, vitamin E, energy, protein, carbohydrate, saturated fatty acids, monounsaturated fatty acids, polyunsaturated fatty acids, and caffeine. In our study, food folate is the form that occurs naturally in food sources. Folic acid is presented in fortified foods. Total folate includes both food folate and folic acid. Folate is the dietary folate equivalent (DFE) conversion that reflects the differential bioavailability. The recommended dietary allowance (RDA) is the intake level sufficient to satisfy the needs of nearly all healthy individuals in a group. For US adults, the RDA for folate was 400 mcg DFE per day, and for vitamin B12, was 2.4 mcg/day for males and females aged 20 years and above. RDAs for vitamin B6 were 1.3 mg/day for males aged 20–50; 1.7 mg/day for males aged 51 and over; 1.3 mg/day for females aged 20–50; and 1.5 mg/day for females aged 51 and over. The patients were divided into two separate groups according to their RDAs for folate (folate: <400 mcg DFE per day and ≥400 mcg DFE per day). In addition, we also divided participants into five groups according to quintiles each for the intakes of folate, vitamin B6, and vitamin B12.

Serum concentrations of UA were detected on a Beckman UniCel^®^ DxC800 Synchron (Beckman Coulter, Inc., Brea, CA, USA) or a Beckman Synchron LX20 (Beckman Coulter, Inc., Brea, CA, USA) after oxidation of UA by uricase to form allantoin and H_2_O_2_. HU was defined as serum UA ≥7.0 mg/dL in males and ≥6.0 mg/dL in females. Sociodemographic characteristics included age, race/ethnicity, marital status, and education. Race/ethnicity was defined as non-Hispanic white, non-Hispanic black, Mexican American, and others. Marital status was classified into married or living with partner, and living alone. Educational background was grouped into above high school, high school graduation/general educational development (GED), and less than high school. Physical examinations, such as weight, height, and blood pressure, were conducted following standardized protocol. Body mass index (BMI) was calculated as weight divided by height^2^ (kg/m^2^). Hypertension was identified as systolic blood pressure ≥140 mm Hg, or diastolic blood pressure ≥90 mm Hg. Smoking status was grouped as never, current, and former smoker, and participants were divided into never drinker, current drinker, and former drinker, according to alcohol intake. Diabetes status was obtained from self-report. Other covariates included serum total cholesterol (STC), glucose, and serum triglycerides (STG).

### 2.3. Statistical Analyses

All statistical analyses were performed using SAS version 9.4 (SAS Institute Inc., Cary, NC, USA). The continuous variables were described by median and interquartile range, depending on the skewed distributed data. The categorical variables were expressed as percentage. Wilcoxon signed-rank test was used to compare the intake of folate, vitamin B6, and vitamin B12 separately with the population RDAs. Differences between continuous variables were assessed using the Wilcoxon rank-sum test. Differences between categorical variables were evaluated by the chi-square test and multiple comparisons based on Bonferroni correction. Multivariable logistic regression analysis was used to estimate the odds ratio (OR) and 95% confidence interval (CI) of HU according to the folate, vitamin B6, and vitamin B12 intake quintile for males and females separately. Model 1 adjusted only for age and race/ethnicity. Model 2 additionally controlled for smoking status, drinking status, educational background, marital status, hypertension status, and diabetes status, based on model 1. Model 3 further adjusted for energy intake, protein intake, carbohydrate intake, vitamin E intake, vitamin B6 intake, vitamin B12 intake, saturated fatty acids intake, monounsaturated fatty acids intake, polyunsaturated fatty acids intake, caffeine intake, STC, glucose, BMI, and STG based on model 2. Model 4 adjusted for folate intake instead of vitamin B6 intake, based on model 3. Model 5 adjusted for folate intake instead of vitamin B12 intake based on model 3. The statistical significance level was set as *p* value <0.05 (two-sided), and *p* < 0.0125 (0.05/4), *p* < 0.0167 (0.05/3), *p* < 0.025 (0.05/2) were considered as statistically significant after Bonferroni adjustment for multiple comparisons.

## 3. Results

The daily folate intake was 511 mcg DFE (342–752 DFE mcg) for males aged 20–85 years, higher than their RDAs (400 mcg DFE per day for males and females aged 20 years and above), and 395 DFE mcg (264–573 DFE mcg) for females aged 20–85 years, lower than their RDA. The daily vitamin B12 intake was 4.65 mcg (2.65–7.57 mcg) for males and 3.26 mcg (1.82–5.29 mcg) for females aged 20–85 years, higher than their respective RDAs (2.40 mcg/day for males and females aged 20 years and above). The daily vitamin B6 intake was 2.16 mg (1.49–3.08 mg) for males and 2.11 mg (1.47–2.11 mg) for females aged 20–50 years, higher than their respective RDAs (1.3 mg/day for males and females aged 20–50 years); 1.85 mg (1.27–2.60 mg) for males aged 51–85 years, higher than their RDA (1.7 mg/day for males aged 51 years and above) and 1.43 mg (0.98–2.01 mg) for females aged 51–85 years, as shown in Table 1. The characteristics of the participants, according to those consuming less than the RDAs of folate intake (DFE), and those consuming the RDA or greater, for both sexes, are shown in Table 2. For males, except for weight (*p* = 0.9471) and STC (*p* = 0.2296), other indicators were all significantly different between the two groups, according to folate intake. Participants with daily folate intakes of 400 mcg DFE or greater, were less likely to be non-Hispanic black, less than high school-educated, living alone, former drinking, and current smoking, and were more likely to have lower BMI, glucose, and UA, and were less likely to have hypertension, diabetes, and HU, compared with those consuming less than 400 mcg folate daily. For females, except for STG (*p* = 0.5920), other indicators were all significantly different between the two groups, according to folate intake. Compared to those participants consuming less than 400 mcg DFE daily, those consuming 400 mcg DFE or greater, daily, were less likely to be non-Hispanic black, high school- or GED-educated, less than high school-educated, living alone, currently smoking, and were less likely to have hypertension, diabetes, and HU, and have lower weight, BMI, STC, glucose, and UA.

The results comparing the B-vitamin intake indicators between HU and non-HU are shown in Table 3. All the B-vitamin intake indicators were significantly different between HU and non-HU for males and females. Compared to the participants without HU, participants with HU had lower intakes of total folate, folic acid, food folate, folate (DFE), vitamin B6, and vitamin B12 for both sexes.

The correlation between folate intake and HU was examined by multivariable model, as provided in Table 4. The results suggested an inverse relationship between folate intake and HU in our cross-sectional study, after controlling for age, race/ethnicity, smoking status, drinking status, education background, marital status, hypertension status, and diabetes status, energy intake, protein intake, carbohydrate intake, vitamin E intake, vitamin B6 intake, vitamin B12 intake, saturated fatty acids intake, monounsaturated fatty acids intake, polyunsaturated fatty acids intake, caffeine intake, STC, glucose, BMI, and STG. In males, there was an inverse trend between higher total folate intake and risk of HU (*p* for trend <0.0001; Table 4), compared with the lowest quintile (Q1; consuming less than 248 mcg/day), and adjusted ORs of HU in Q2 to Q5 were 0.87 (95% CI, 0.75–0.99), 0.84 (95% CI, 0.73–0.97), 0.73 (95% CI, 0.62–0.85), and 0.64 (95% CI, 0.53–0.77), respectively. In females, compared with Q1 (respondents consuming less than 192 mcg total folate daily), the ORs of HU in Q2 to Q4 of the total folate intake were 0.82 (95% CI, 0.70–0.97), 0.82 (95% CI, 0.69–0.97), and 0.79 (95% CI, 0.65–0.95), respectively. The OR was 0.84 (95% CI, 0.67−1.04) for Q5, and *p* for trend was 0.0767.

In males, compared with Q1 (respondents consuming less than 70 mcg folic acid daily), the adjusted ORs of HU were 0.78 (95% CI, 0.68–0.91) for Q4 (consuming 195–311 mcg folic acid daily), 0.75 (95% CI, 0.64–0.89) for Q5 (consuming 312 mcg or greater), and a *p* for trend of <0.0001. No significant relationship between folic acid intake and HU was found in females.

Compared to Q1, adjusted ORs in Q2 to Q5 of the food folate intake were 0.79 (95% CI, 0.69–0.91), 0.82 (95% CI, 0.71–0.95), 0.83 (95% CI, 0.71–0.97), and 0.70 (95% CI, 0.59–0.84), respectively, and the *p* for trend was 0.0018 in males. In females, compared to Q1, the adjusted ORs of HU were 0.81 (95% CI, 0.67–0.97) for Q4 (those consuming 192–261 mcg food folate daily), 0.80 (95% CI, 0.65–0.99) for Q5 (those consuming 262 mcg or greater), and the *p* for trend was 0.0132.

In men, compared to Q1, adjusted ORs in Q2 to Q5 of the folate (DFE) intake were 0.84 (95% CI, 0.73–0.96), 0.84 (95% CI, 0.73–0.97), 0.72 (95% CI, 0.62–0.84), and 0.64 (95% CI, 0.53–0.77), respectively (*p* for trend <0.0001). In women, compared to Q1, adjusted ORs in Q2 to Q4 of the folate (DFE) intake were 0.84 (95% CI, 0.71–0.99), 0.81 (95% CI, 0.68–0.96) and 0.82 (95% CI, 0.68–0.99), respectively. The OR was 0.87 (95% CI, 0.70−1.07) for Q5, and the *p* for trend was 0.1475.

We did not observe associations between higher vitamin B6 intake and risk of HU in both males and females after adjusting for potential confounders, as presented in Table 5. As shown in Table 6, an inverse association between vitamin B12 intake and HU existed in males. Compared with Q1 (respondents consuming less than 2.28 mcg vitamin B12 daily), the adjusted ORs of HU were 0.79 (95% CI, 0.67–0.93) for Q4 (those consuming 5.59–8.43 mcg vitamin B12 daily), 0.77 (95% CI, 0.64–0.93) for Q5 (those consuming 8.44 mcg or greater), and the *p* for trend was 0.0013, but no significant relationship between vitamin B12 intake and HU in females was found in this study.

## 4. Discussion

To the best of our knowledge, this is the first study to show an association between intakes of folate, vitamin B6, and vitamin B12 with HU, and the largest population-based study using a nationally representative sample in the US. As such, our results must be interpreted cautiously. In this large population-based cross-sectional study in US adults, we observed inverse associations between intakes of total folate, folic acid (Q1 vs Q4, Q5), food folate, and folate (DFE) in men, and intake of vitamin B12 (Q1 vs Q4, Q5), but not vitamin B6, was also inversely related to risk of HU in men. We observed a lower risk of HU with higher intakes of total folate (Q1 vs Q2–Q4), food folate (Q1 vs Q4, Q5), and folate (DFE) (Q1 vs Q2–Q4) in women, and we did not observe associations with intakes of folic acid, vitamin B6, or vitamin B12 in women.

Although the underlying mechanisms of the association between the intakes of folate, vitamin B6, and vitamin B12 with HU are largely unknown, several hypotheses have been proposed. First, folate and its derivatives may inactivate XOR, which is essential for the oxidation from hypoxanthine to xanthine, and from xanthine to UA [8,9]. Folate inhibits XOR by slow binding with high affinity at the molybdenum site, the site of purine interaction, and folic acid disrupts the interaction of the enzyme with xanthine and hypoxanthine [8,9]. In addition, studies have suggested that folate is a potent Hcy-lowering agent [26], and lower Hcy levels have been reported to be associated with a lower risk of HU [14,15]. One possible explanation is that chronic elevation in Hcy results in parallel increases in intracellular *S*-adenosylhomocysteine [27]. As a potent inhibitor for most *S*-adenosylmethionine-dependent methyltransferases, *S*-adenosylhomocysteine may induce marked DNA damage and release purine nucleotides [28,29]. The catabolism of purine nucleotides ultimately results in the production of UA [30]. Therefore, lowering the Hcy level is likely to result in a beneficial effect in lowering serum UA levels, and lowering Hcy levels can be achieved by increasing intakes of folate, vitamin B6, and vitamin B12 [16].

Our study showed that increased intakes of total folate, folic acid, food folate, folate (DFE), and vitamin B12 may decrease the risks of HU in males. Consistent with this, a randomized, double-blind, actively controlled trial showed that compared with enalapril alone, the combination of enalapril and folic acid significantly reduced the magnitude of the increase in UA concentrations in hypertensive adults [17]. A cross-sectional study found that vegetables and fruit, which are rich in dietary fiber, folate, and vitamin C, appear to be protective against gout [18]. A study by Oster suggested that very high doses of supplemental folate may be beneficial in preventing the incidence and recurrence of gout [19]. Adding folic acid and eicosapentaenoic acid to the diet may relieve symptoms of gouty arthritis [31]. However, other intervention studies that used folic acid supplementation failed to lower blood UA concentrations [20]. Pteroylglutamic acid, administered in doses up to 1000 mg, did not significantly lower the serum urate concentration nor decrease urinary urate concentration or total oxypurine excretion in HU subjects [21]. Our study also did not observe associations with intakes of folic acid in females.

Numerous studies have found HU may be linked to various food. The intake of soy products [32] and dairy product [33] are inversely associated with HU. The consumption of nuts, legumes, and whole grains could effectively lower the risk of gout [34]. Vegetables and fruit, which are rich in folate, dietary fiber, and vitamin C, might be useful for protection against gout [18]. Similarly, our study showed that increasing the intake of naturally occurring folate from food sources may decrease the risk of HU in both males and females. Folate occurs naturally in a wide variety of foods. Fruits, fruit juices, and vegetables (especially dark green leafy vegetables) are good dietary sources of folate. Spinach, asparagus, yeast, and Brussels sprouts are among the foods with the highest levels of folate. A variety of protein foods, including lean meats, poultry, eggs, and soy products, are all rich in folate. Legumes (beans and peas), nuts, and seeds also have folate [35]. Additionally, bread, rice, flour, cereal, cornmeal, pasta, and other grain products are fortified with folic acid in the US [36].

Our study has several strengths. Firstly, this is the first study, to our knowledge, to assess the association between intakes of folate, vitamin B6, vitamin B12, and the risk of HU, and the largest population-based study using a nationally representative sample among US adults. Secondly, we adjusted for a wide range of potential confounding variables. Thirdly, the exactitude and efficacy of the data acquired was improved using trained staff, following standardized protocols to assess the main information of study subjects and conduct interviews.

There are also several limitations to our study. First, our study used a cross-sectional design, which limited the definition causality or the temporal relationship between B-vitamin intakes and HU. Further prospective longitudinal investigations are important to support the conclusions. Second, although we adjusted for several major covariates in our multivariable models, the associations reported may partially result from other unobserved and unknown confounding variables, and residual confounding. Third, nutrient intake levels were assessed by 24-h dietary recall method, which may not precisely reflect the long-term B-vitamin intake status. However, compared with food frequency questionnaires, 24-h recall supplies more detail on the kinds and amounts of food eaten, and diminishes the risk of underestimating or overestimating the B-vitamin intake level. Fourth, the study lacked blood concentration measurements and other biomarker measures; however, blood levels may not comprehensively reveal the nutritional condition [37]. Fifth, our study was restricted to persons of European ancestry, limiting the generalizability of results. Finally, further studies are needed to investigate the underlying mechanisms of the reported association.

## 5. Conclusions

Our findings indicated the intakes of total folate, folic acid, food folate, folate (DFE), and vitamin B12, but not vitamin B6, were inversely related to risk of HU in males. We observed a lower risk of HU with higher intakes of total folate, food folate, folate (DFE) in females, and no association between intakes of folic acid, vitamin B6, vitamin B12, and the risk of HU in females, independent of some major confounding factors.

## Figures and Tables

**Table 1:** B-vitamin intake among US adults (>19 years) in NHANES 2001–2014.

**Age (Years)**	**RDAs for Folate, DFE (mcg/day)**	**Folate, DFE (mcg/day)**	***p***
20–85 ^a^	400.00	511.00 (342.00, 752.00)	<0.0001
20–85 ^b^	400.00	395.00 (264.00, 573.00)	<0.0001
**Age (Years)**	**RDAs for Vitamin B6 (mg/day)**	**Vitamin B6 (mg/day)**	***p***
20–50 ^a^	1.30	2.16 (1.49, 3.08)	<0.0001
20–50 ^b^	1.30	2.11 (1.47, 2.11)	<0.0001
51–85 ^a^	1.70	1.85 (1.27, 2.60)	<0.0001
51–85 ^b^	1.50	1.43 (0.98, 2.01)	0.8645
**Age (Years)**	**RDAs for Vitamin B12 (mcg/day)**	**Vitamin B12 (mcg/day)**	***p***
20–85 ^a^	2.40	4.65 (2.65, 7.57)	<0.0001
20–85 ^b^	2.40	3.26 (1.82, 5.29)	<0.0001

^a^ Male; ^b^ Female.

**Table 2:** Characteristics of the participants according to intake of folate (DFE).

Characteristic	Male		*p*	Female		*p*
	Folate Intake <400 (mcg/day) (*n* = 4357)	Folate Intake ≥400 (mcg/day) (*n* = 8400)		Folate Intake <400 (mcg/day) (*n* = 6200)	Folate Intake ≥400 (mcg/day) (*n* = 6018)	
Age (years)	50.00 (34.00, 64.00)	45.00 (32.00, 60.00)	<0.0001	49.00 (35.00, 63.00)	47.00 (34.00, 62.00)	0.0002
Race/ethnicity (*n*, %)			<0.0001			<0.0001
Non-Hispanic white	1947 (44.69)	4265 (50.77)	0.0001 ^c^	2924 (47.16)	3030 (50.35)	0.0386 ^c^
Non-Hispanic black	1048 (24.05)	1381 (16.44)	<0.0001 ^c^	1413 (22.79)	954 (15.85)	<0.0001 ^c^
Mexican American	781 (17.93)	1518 (18.07)	0.8651 ^c^	1009 (16.27)	1037 (17.23)	0.2315 ^c^
Others ^a^	581 (13.33)	1236 (14.71)	0.0665 ^c^	854 (13.77)	997 (16.57)	0.0002 ^c^
Education background (*n*, %)			<0.0001			<0.0001
Above high school	1895 (43.49)	4437 (52.82)	<0.0001 ^d^	3085 (49.76)	3421 (56.85)	<0.0001 ^d^
High school or GED ^b^	1098 (25.20)	1968 (23.43)	0.0828 ^d^	1481 (23.89)	1271 (21.12)	0.0036 ^d^
Less than high school	1364 (31.31)	1995 (23.75)	<0.0001 ^d^	1634 (26.35)	1326 (22.03)	<0.0001 ^d^
Marital status (*n*, %)			<0.0001			<0.0001
Married or living with partner	2778 (63.76)	5692 (67.76)	0.0406 ^e^	3291 (53.08)	3496 (58.09)	0.0029 ^e^
Living alone	1579 (36.24)	2708 (32.24)	0.0014 ^e^	2909 (46.92)	2522 (41.91)	0.0005 ^e^
Drinking status (*n*, %)			0.0004			0.0038
Never	308 (7.07)	600 (7.14)	0.8862 ^d^	1217 (19.63)	1130 (18.78)	0.3251 ^d^
Current	3609 (82.83)	7127 (84.85)	0.3856 ^d^	3741 (60.34)	3800 (63.14)	0.1208 ^d^
Former	440 (10.10)	673 (8.01)	0.0003 ^d^	1242 (20.03)	1088 (18.08)	0.0235 ^d^
Smoking status (*n*, %)			<0.0001			<0.0001
Never	1818 (41.73)	3905 (46.49)	0.0015 ^d^	3677 (59.31)	3848 (63.94)	0.0103 ^d^
Current	1269 (29.13)	2083 (24.80)	<0.0001 ^d^	1341 (21.63)	977 (16.23)	<0.0001 ^d^
Former	1270 (29.15)	2412 (28.71)	0.7031 ^d^	1182 (19.06)	1193 (19.82)	0.3838 ^d^
Weight (kg)	83.70 (72.90, 96.50)	83.60 (73.40, 95.90)	0.9471	72.10 (61.40, 85.95)	70.90 (60.80, 84.30)	0.0012
Height (cm)	173.90 (168.90, 179. 00)	175.10 (169.80, 180.30)	<0.0001	160.80 (155.70, 165.50)	161.50 (156.60, 166.10)	<0.0001
BMI (kg/m^2^)	27.72 (24.70, 31.32)	27.44 (24.38, 30.81)	0.0003	28.07 (24.07, 33.07)	27.30 (23.43, 32.38)	<0.0001
Hypertension status (*n*, %)	1049 (24.08)	1685 (20.06)	<0.0001	1340 (21.61)	1196 (19.87)	0.0178
Diabetes status (*n*, %)	451 (10.35)	734 (8.74)	0.0029	649 (10.47)	521 (8.66)	0.0007
HU (*n*, %)	1065 (24.44)	1627 (19.37)	<0.0001	1099 (17.73)	846 (14.06)	<0.0001
Energy intake (kcal/day)	1743.00 (1327.00, 2231.00)	2670.50 (2079.00, 3396.00)	<0.0001	1415.00 (1076.00, 1810.00)	2000.50 (1587.00, 2513.00)	<0.0001
Protein intake (gm/day)	67.03 (47.94, 89.55)	100.09 (76.10, 131.32)	<0.0001	53.00 (38.36, 70.15)	74.96 (57.37, 94.95)	<0.0001
Carbohydrate intake (gm/day)	195.95 (143.39, 262.95)	318.35 (245.06, 412.90)	<0.0001	169.54 (125.50, 222.07)	253.86 (200.89, 318.50)	<0.0001
Vitamin B6 Intake (mg/day)	1.43 (0.98, 2.01)	2.36 (1.72, 3.26)	<0.0001	1.12 (0.77, 1.54)	1.86 (1.38, 2.48)	<0.0001
Vitamin B12 intake (mcg/day)	3.16 (1.79, 5.08)	5.59 (3.40, 8.64)	<0.0001	2.40 (1.39, 3.88)	4.31 (2.67, 6.59)	<0.0001
Vitamin E Intake (mg/day)	4.70 (3.10, 6.96)	8.38 (5.65, 12.16)	<0.0001	4.33 (2.83, 6.40)	7.27 (4.98, 10.35)	<0.0001
Caffeine intake (mg/day)	106.00 (19.00, 234.00)	128.00 (30.00, 260.50)	<0.0001	87.00 (10.00, 191.00)	97.00 (14.00, 208.00)	0.0002
Saturated fatty acids intake (gm/day)	19.91 (12.69, 29.45)	30.71 (20.51, 43.74)	<0.0001	16.79 (10.92, 24.30)	22.62 (15.23, 32.75)	<0.0001
Monounsaturated fatty acids (gm/day)	23.34 (15.33, 33.25)	35.16 (24.16, 49.30)	<0.0001	18.85 (12.47, 26.75)	25.48 (17.51, 36.39)	<0.0001
Polyunsaturated fatty acids (gm/day)	12.71 (7.71, 18.86)	20.25 (13.48, 28.73)	<0.0001	11.07 (6.96, 16.73)	15.81 (10.75, 23.15)	<0.0001
STC (mg/dL)	193.00 (166.00, 221.00)	192.00 (166.00, 219.00)	0.2296	198.00 (172.00, 227.00)	195.00 (170.00, 223.00)	0.0004
Glucose (mg/dL)	94.00 (87.00, 104.00)	93.00 (86.00, 103.00)	<0.0001	91.00 (84.00, 101.00)	90.00 (83.00, 100.00)	0.0249
STG (mg/dL)	124.00 (81.00, 194.00)	128.00 (84.00, 202.00)	0.0069	109.00 (74.00, 163.00)	110.00 (74.00, 168.00)	0.5920
UA (mg/dL)	6.10 (5.30, 6.90)	5.90 (5.10, 6.70)	<0.0001	4.70 (4.00, 5.60)	4.60 (3.90, 5.40)	<0.0001

^a^ Other Hispanics and other races, including multi-racial participants; ^b^ General educational development; ^c^ Statistically significant after Bonferroni adjustment (0.05/4 = 0.0125); ^d^ Statistically significant after Bonferroni adjustment (0.05/3 = 0.0167); ^e^ Statistically significant after Bonferroni adjustment (0.05/2 = 0.025).

**Table 3:** B-vitamin intakes characteristics of participants with or without HU.

Characteristic	Male		*p*	Female		*p*
	Non-HU (*n* = 10,065)	HU (*n* = 2692)		Non-HU (*n* = 10,273)	HU (*n* = 1945)	
Total folate intake (mcg/day)	408.00 (279.00, 582.00)	368.00 (250.00, 523.50)	<0.0001	313.00 (217.00, 443.00)	285.00 (190.00, 406.00)	<0.0001
Folic acid intake (mcg/day)	162.00 (86.00, 281.00)	137.00 (71.00, 240.00)	<0.0001	121.00 (63.00, 206.00)	109.00 (58.00, 188.00)	<0.0001
Food folate intake (mcg/day)	218.00 (149.00, 315.00)	205.00 (136.00, 297.00)	<0.0001	169.00 (113.00, 243.00)	153.00 (104.00, 223.00)	<0.0001
Folate intake, DFE (mcg/day)	526.00 (350.00, 772.00)	469.50 (312.00, 684.00)	<0.0001	401.00 (269.00, 580.00)	361.00 (238.00, 532.00)	<0.0001
Vitamin B6 intake (mg/day)	2.03 (1.40, 2.89)	1.99 (1.36, 2.81)	0.0219	1.46 (1.00, 2.07)	1.39 (0.94, 1.98)	<0.0001
Vitamin B12 intake (mcg/day)	4.74 (2.70, 7.69)	4.32 (2.50, 6.98)	<0.0001	3.29 (1.84, 5.37)	3.06 (1.71, 4.97)	<0.0001

**Table 4:** Adjusted odds ratios of HU among participants associated with folate intake.

		**Total Folate Intake (mcg/day)**					***p* for Trend**
		**Q1 (≤247)** ** (*n* = 2562** **)**	**Q2 (248–347)** ** (*n* = 2567** **)**	**Q3 (348–457)** ** (*n* = 2547** **)**	**Q4 (458–620)** ** (*n* = 2535** **)**	**Q5 (≥621)** ** (*n* = 2546** **)**	
Male (*n* = 12,757)	Model 1	Reference	0.82 (0.72, 0.93)	0.81 (0.71, 0.92)	0.72 (0.63, 0.82)	0.55 (0.48, 0.64)	<0.0001
	Model 2	Reference	0.80 (0.71, 0.91)	0.79 (0.69, 0.90)	0.69 (0.61, 0.79)	0.53 (0.46, 0.61)	<0.0001
	Model 3	Reference	0.87 (0.75, 0.99)	0.84 (0.73, 0.97)	0.73 (0.62, 0.85)	0.64 (0.53, 0.77)	<0.0001
		**Q1 (≤191)** ** (*n* = 2454** **)**	**Q2 (192–268)** ** (*n* = 2454** **)**	**Q3 (269–353)** ** (*n* = 2442** **)**	**Q4 (354–476)** ** (*n* = 2426** **)**	**Q5 (≥477)** ** (*n* = 2442** **)**	
Female (*n* = 12,218)	Model 1	Reference	0.78 (0.67, 0.91)	0.722 (0.62,0.84)	0.70 (0.60, 0.81)	0.64 (0.55, 0.75)	<0.0001
	Model 2	Reference	0.78 (0.67, 0.90)	0.73 (0.63, 0.86)	0.71 (0.61, 0.83)	0.65 (0.56, 0.77)	<0.0001
	Model 3	Reference	0.82 (0.70, 0.97)	0.82 (0.69, 0.97)	0.79 (0.65, 0.95)	0.84 (0.67, 1.04)	0.0767
		**Folic Acid Intake(mcg/day)**					
		**Q1 (≤69)** ** (*n* = 2585)**	**Q2 (70–125)** ** (*n* = 2523)**	**Q3 (126–194)** ** (*n* = 2573)**	**Q4 (195–311)** ** (*n* = 2532)**	**Q5 (≥312)** ** (*n* = 2544)**	
Male (*n* = 12,757)	Model 1	Reference	0.89 (0.78, 1.01)	0.82 (0.72, 0.93)	0.67 (0.58, 0.77)	0.58 (0.51, 0.67)	<0.0001
	Model 2	Reference	0.89 (0.78, 1.01)	0.80 (0.70, 0.91)	0.66 (0.57, 0.75)	0.56 (0.49, 0.65)	<0.0001
	Model 3	Reference	0.95 (0.83, 1.09)	0.89 (0.77, 1.02)	0.78 (0.68, 0.91)	0.75 (0.64, 0.89)	<0.0001
		**Q1 (≤52)** ** (*n* = 2482** **)**	**Q2 (53–94) ** **(*n* = 2429)**	**Q3 (95–147) ** **(*n* = 2456)**	**Q4 (148–230) ** **(*n* = 2417)**	**Q5 (≥231)** **(*n* = 2434)**	
Female (*n* = 12,218)	Model 1	Reference	1.00 (0.86, 1.17)	0.95 (0.82, 1.11)	0.90 (0.77, 1.05)	0.85 (0.72, 0.99)	<0.0001
	Model 2	Reference	0.99 (0.85, 1.16)	0.95 (0.81, 1.10)	0.89 (0.76, 1.05)	0.85 (0.72, 0.99)	0.0169
	Model 3	Reference	1.012 (0.86, 1.19)	1.00 (0.84, 1.18)	0.97 (0.82, 1.16)	1.04 (0.86, 1.25)	0.9137
		**Food Folate Intake(mcg/day)**					
		**Q1 (≤132.0)** ** (*n* = 2584** **)**	**Q2 (132.1–185.0)** ** (*n* = 2529** **)**	**Q3 (185.1–247.0)** ** (*n* = 2551** **)**	**Q4 (247.1–339.8)** ** (*n* = 2541** **)**	**Q5 (≥339.9)** ** (*n* = 2552** **)**	
Male (*n* = 12,757)	Model 1	Reference	0.78 (0.68, 0.89)	0.78 (0.69, 0.90)	0.82 (0.72, 0.94)	0.68 (0.60, 0.78)	<0.0001
	Model 2	Reference	0.76 (0.66, 0.86)	0.75 (0.66, 0.86)	0.79 (0.69, 0.90)	0.66 (0.57, 0.75)	<0.0001
	Model 3	Reference	0.79 (0.69, 0.91)	0.82 (0.71, 0.95)	0.83 (0.71, 0.97)	0.70 (0.59, 0.84)	0.0018
		**Q1 (≤101)** ** (*n* = 2492** **)**	**Q2 (102–143)** ** (*n* = 2403** **)**	**Q3 (144–191)** ** (*n* = 2468** **)**	**Q4 (192–261)** ** (*n* = 2420** **)**	**Q5 (≥262)** ** (*n* = 2435** **)**	
Female (*n* = 12,218)	Model 1	Reference	0.88 (0.76, 1.03)	0.81 (0.70, 0.94)	0.73 (0.62, 0.85)	0.65 (0.55, 0.76)	<0.0001
	Model 2	Reference	0.89 (0.76, 1.04)	0.83 (0.71, 0.96)	0.74 (0.64, 0.87)	0.67 (0.57, 0.79)	<0.0001
	Model 3	Reference	0.93 (0.79, 1.10)	0.89 (0.75, 1.05)	0.81 (0.67, 0.97)	0.80 (0.65, 0.99)	0.0132
		**Folate Intake, DFE (mcg/day)**					
		**Q1 (≤310)** ** (*n* = 2556** **)**	**Q2 (311–440)** **(*n* = 2557)**	**Q3 (441–593) ** **(*n* = 2556)**	**Q4 (594–825) ** **(*n* = 2538)**	**Q5 (≥826) ** **(*n* = 2550)**	
Male (*n* = 12,757)	Model 1	Reference	0.82 (0.72, 0.93)	0.81 (0.72, 0.93)	0.68 (0.59, 0.78)	0.55 (0.48, 0.63)	<0.0001
	Model 2	Reference	0.80 (0.70, 0.91)	0.79 (0.69, 0.90)	0.65 (0.57, 0.75)	0.53 (0.46, 0.61)	<0.0001
	Model 3	Reference	0.84 (0.73, 0.96)	0.84 (0.73, 0.97)	0.72 (0.62, 0.84)	0.64 (0.53, 0.77)	<0.0001
		**Q1 (≤239)** ** (*n* = 2448** **)**	**Q2 (240–340)** ** (*n* = 2442** **)**	**Q3 (341–455)** ** (*n* = 2446** **)**	**Q4 (456–629)** ** (*n* = 2443** **)**	**Q5 (≥630)** ** (*n* = 2439** **)**	
Female (*n* = 12,218)	Model 1	Reference	0.78 (0.67, 0.91)	0.72 (0.62, 0.84)	0.71 (0.61, 0.83)	0.67 (0.57, 0.78)	<0.0001
	Model 2	Reference	0.78 (0.67, 0.90)	0.73 (0.62, 0.85)	0.73 (0.62, 0.85)	0.67 (0.57, 0.79)	<0.0001
	Model 3	Reference	0.84 (0.71, 0.99)	0.81 (0.68, 0.96)	0.82 (0.68, 0.99)	0.87 (0.70, 1.07)	0.1475

Model 1 adjusted for age, race/ethnicity; Model 2 adjusted for age, race/ethnicity, smoking status, drinking status, education background, marital status, hypertension status, and diabetes status; Model 3 adjusted for age, race/ethnicity, smoking status, drinking status, education background, marital status, hypertension status, and diabetes status, energy intake, protein intake, carbohydrate intake, vitamin E intake, vitamin B6 intake, vitamin B12 intake, saturated fatty acids intake, monounsaturated fatty acids intake, polyunsaturated fatty acids intake, caffeine intake, STC, glucose, BMI, and STG.

**Table 5:** Adjusted odds ratios of HU among participants associated with vitamin B6 intake.

		Vitamin B6 Intake (mg/day)					*p* for Trend
		**Q1 (≤1.253)** ** (*n* = 2552** **)**	**Q2 (1.254–1.758)** ** (*n* = 2554** **)**	**Q3 (1.759–2.307)** ** (*n* = 2549** **)**	**Q4 (2.308–3.141)** ** (*n* = 2550** **)**	**Q5 (≥3.142)** ** (*n* = 2552** **)**	
Male (*n* = 12,757)	Model 1 Model 2 Model 4	Reference Reference Reference	1.02 (0.89, 1.16) 0.99 (0.87, 1.14) 1.06 (0.92, 1.22)	1.08 (0.95, 1.24) 1.05 (0.92, 1.20) 1.16 (0.99, 1.35)	0.92 (0.80, 1.05) 0.88 (0.76, 1.01) 0.99 (0.84, 1.17)	0.90 (0.79, 1.04) 0.88 (0.76, 1.01) 1.11 (0.91, 1.36)	0.0505 0.0141 0.5782
		**Q1 (≤0.896)** ** (*n* = 2452** **)**	**Q2 (0.897–1.263)** ** (*n* = 2437** **)**	**Q3 (1.264–1.657)** ** (*n* = 2447** **)**	**Q4 (1.658–2.240)** ** (*n* = 2441** **)**	**Q5 (≥2.241)** ** (*n* = 2441** **)**	
Female (*n* = 12,218)	Model 1 Model 2 Model 4	Reference Reference Reference	0.94 (0.81, 1.10) 0.96 (0.82, 1.12) 0.98 (0.83, 1.16)	0.79 (0.67, 0.92) 0.80 (0.68, 0.94) 0.85 (0.71, 1.02)	0.86 (0.74, 1.00) 0.88 (0.75, 1.03) 0.98 (0.81, 1.18)	0.82 (0.70, 0.96) 0.85 (0.73, 1.00) 1.03 (0.82, 1.30)	0.0064 0.0226 0.9808

Model 1 adjusted for age, race/ethnicity; Model 2 adjusted for age, race/ethnicity, smoking status, drinking status, education background, marital status, hypertension status, and diabetes status; Model 4 adjusted for age, race/ethnicity, smoking status, drinking status, education background, marital status, hypertension status, and diabetes status, energy intake, protein intake, carbohydrate intake, vitamin E intake, folate intake, vitamin B12 intake, saturated fatty acids intake, monounsaturated fatty acids intake, polyunsaturated fatty acids intake, caffeine intake, STC, glucose, BMI, and STG.

**Table 6:** Adjusted odds ratios of HU among participants associated with vitamin B12 intake.

		Vitamin B12 Intake (mcg/day)					*p* for Trend
		**Q1 (≤2.27)** ** (*n* = 2552** **)**	**Q2 (2.28–3.79)** ** (*n* = 2560** **)**	**Q3 (3.80–5.58)** ** (*n* = 2547** **)**	**Q4 (5.59–8.43)** ** (*n* = 2549** **)**	**Q5 (≥8.44)** ** (*n* = 2549** **)**	
Male (*n* = 12,757)	Model 1 Model 2 Model 5	Reference Reference Reference	0.98 (0.86, 1.11) 0.96 (0.84, 1.10) 0.95 (0.82, 1.09)	0.94 (0.82, 1.07) 0.93 (0.81, 1.06) 0.94 (0.81, 1.09)	0.79 (0.69, 0.91) 0.77 (0.67, 0.88) 0.79 (0.67, 0.93)	0.73 (0.64, 0.84) 0.72 (0.63, 0.83) 0.77 (0.64, 0.93)	<0.0001 <0.0001 0.0013
		**Q1 (≤1.56)** ** (*n* = 2450** **)**	**Q2 (1.57–2.64)** ** (*n* = 2446** **)**	**Q3 (2.65–3.91)** ** (*n* = 2443** **)**	**Q4 (3.92–5.93)** ** (*n* = 2441** **)**	**Q5 (≥5.94)** ** (*n* = 2438** **)**	
Female (*n* = 12,218)	Model 1 Model 2 Model 5	Reference Reference Reference	0.89 (0.76, 1.04) 0.90 (0.77, 1.05) 0.89 (0.75, 1.04)	0.94 (0.81, 1.09) 0.94 (0.81, 1.10) 0.93 (0.79, 1.11)	0.82 (0.70, 0.96) 0.84 (0.71, 0.98) 0.85 (0.71, 1.02)	0.76 (0.65, 0.90) 0.78 (0.66, 0.91) 0.83 (0.67, 1.03)	0.0007 0.0019 0.1030

Model 1 adjusted for age, race/ethnicity; Model 2 adjusted for age, race/ethnicity, smoking status, drinking status, education background, marital status, hypertension status, and diabetes status; Model 5 adjusted for age, race/ethnicity, smoking status, drinking status, education background, marital status, hypertension status, and diabetes status, energy intake, protein intake, carbohydrate intake, vitamin E intake, folate intake, vitamin B6 intake, saturated fatty acids intake, monounsaturated fatty acids intake, polyunsaturated fatty acids intake, caffeine intake, STC, glucose, BMI, and STG.
